# Good Visual Outcome Following Corticosteroid Treatment for Compressive Optic Neuropathy Secondary to Sinonasal Carcinoma

**DOI:** 10.7759/cureus.7732

**Published:** 2020-04-18

**Authors:** Amirah Mohammad Razali, Ayesha Mohd Zain, Wan Haslina Bt Wan Abdul Halim, Norshamsiah Md Din

**Affiliations:** 1 Ophthalmology, Universiti Kebangsaan Malaysia Medical Centre, Kuala Lumpur, MYS

**Keywords:** compressive optic neuropathy, sinonasal carcinoma, corticosteroid treatment

## Abstract

Most patients with sinonasal carcinoma present to the otorhinolaryngologist with nasal symptoms. It is however uncommon for them to present with acute visual loss at first presentation. We report a case of compressive optic neuropathy secondary to sinonasal carcinoma, which presented acutely with right eye blurring of vision upon waking up. Computed tomography (CT) of the brain and orbit with contrast showed a locally invasive nasopharyngeal mass extending into the right orbit and cranial fossa. Histopathological examination revealed squamous cell sinonasal carcinoma. Her visual acuity improved with a three-day course of pulsed intravenous methylprednisolone 1 g per day, followed by a gradual tapering dose of oral prednisolone (1 mg/kg/day).

## Introduction

Sinonasal carcinoma is a rare head and neck malignancy. Early symptoms are non-specific with nasal congestion and rhinorrhoea. Thus, presentation is often late, with invasion of surrounding structures such as orbit, skull base and central nervous system. Orbital invasion occurs in 10.6-22.3% via neurovascular structures, preformed pathways such as the orbital fissure, nasolacrimal duct and optic canal, or direct extension through bone [1,2]. Commonest ocular presentations are proptosis and diplopia [1]. Other symptoms include epiphora, periorbital pain and swelling, visual field loss and blurring of vision from optic neuropathy [3].

For sinonasal carcinoma with orbital involvement, the epicentre of the mass is most commonly the nasal cavity. It is then followed by the maxillary sinus, which then invades the anterior skull base and less commonly the pterygopalatine and infratemporal fossa [1]. Optic nerve compression usually occurs at the intra-orbital portion of the optic nerve, but it may also involve the intracranial portion and optic chiasm in cases of aggressive tumours [3,4]. Tumours involving the anterior visual pathway usually present with gradual, progressive blurring of vision [5].

Treatment of compressive optic neuropathy in cases of malignancy can either be surgical decompression, chemo and radiotherapy to shrink the tumour, and also steroid therapy. Pulse intravenous methylprednisolone reduces the tissue oedema, hence reducing the compressive effects of the tumour [6].

## Case presentation

**Figure 1:**
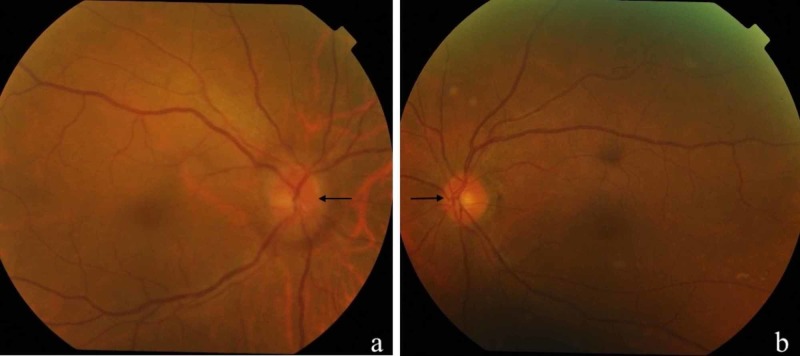
Fundus photo of the right eye showing hyperaemic optic disc with blurred margins (a). The optic disc was normal in the left eye (b).

A 58-years-old lady presented with sudden onset, painless blurring of vision of the right eye upon waking up. She has been having rhinorrhoea for two years with minimal blood-stained nasal discharge for two months. She also had right cheek numbness for three months associated with deficiency of tears from the right eye whenever she cries. She denied constitutional symptoms. On examination, right visual acuity was light perception and left was 6/9. There was a right relative afferent pupillary defect. Anterior segment examination was unremarkable bilaterally with normal intraocular pressure. Fundus examination of right eye revealed a swollen and hyperaemic optic disc with normal macula (Figure 1). The left eye was normal. Extraocular movement was normal with no proptosis.

**Figure 2:**
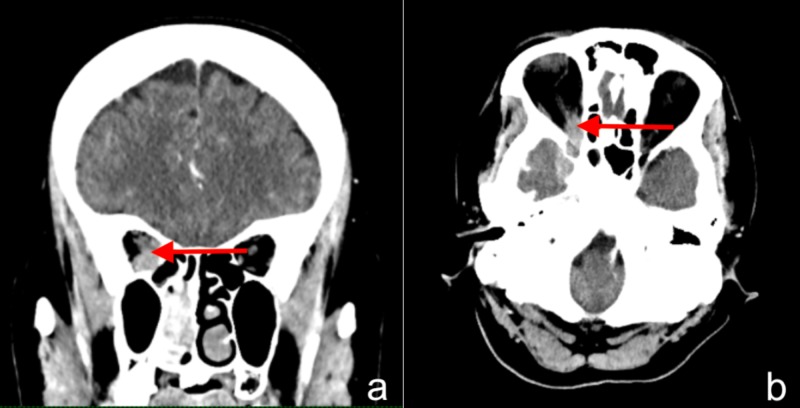
CT scan of orbit showing the mass encasing the right intra-orbital optic nerve at the orbital apex (red arrow) in the coronal view (a) and axial view (b).

CT brain and orbit with contrast revealed a nasopharyngeal mass with the epicentre of mass lying at the right posterior choana with extension to the root of the nasopharynx, right pterygopalatine fossa, orbital fossa and cranial fossa. There was perineural spread along the right inferior orbital fissure and foramen rotundum. The right intra-orbital optic nerve was encased by the mass at the orbital apex (Figure 2). Nasal endoscopy showed a mass at the right middle meatus extending to the nasopharynx. Histopathological examination showed non-keratinizing squamous cell carcinoma. A final diagnosis of sinonasal squamous cell carcinoma (T4b N1 M0) was made.

**Figure 3:**
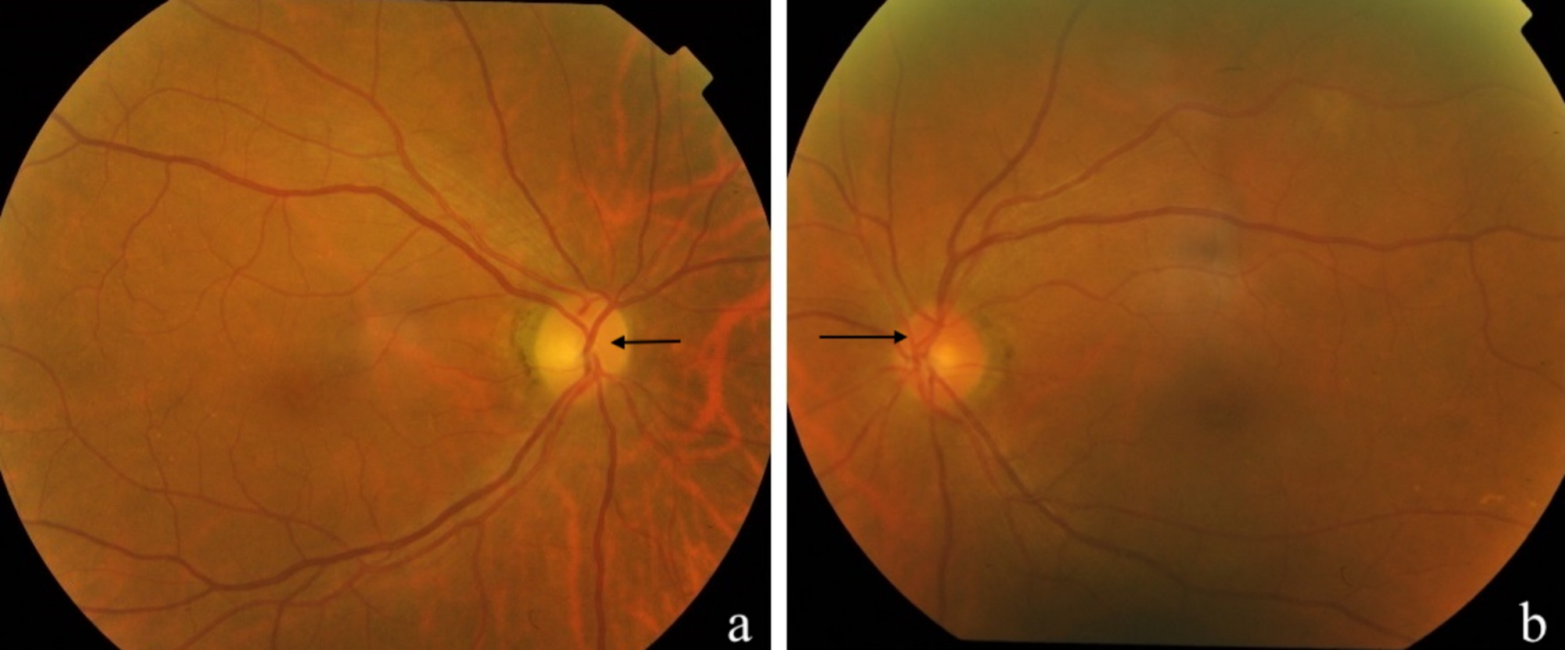
The right optic disc was pale (a) one month after presentation compared to left optic disc (b).

A three-day course of intravenous methylprednisolone 1 g daily was given followed by oral prednisolone 1 mg/kg/day with a slow tapering regime. Right visual acuity improved from light perception to 6/24 at day two of treatment. She also received two cycles of neoadjuvant chemotherapy consisting of intravenous cisplatin and 5-fluorouracil, but unfortunately it was complicated with acute right lower limb ischaemia. Subsequently, palliative radiotherapy with a total dose of 40 Gray in 15 fractions was given. Right visual acuity remained 6/24 six months after presentation with a pale optic disc which developed one month after presentation (Figure 3).

## Discussion

Sinonasal carcinoma is a rare malignancy, comprising only 3% of all head and neck tumours. Relatively higher rates are seen in Asia, especially in Japanese men [7]. The main histological subtypes are squamous cell carcinoma (60-70%) and adenocarcinoma (10-20%) [8]. Tumour growth in an air-filled cavity leads to non-specific symptoms such as rhinorrhoea, nasal congestion, blood stained discharge and anosmia. Hence, patients usually present late with invasion to surrounding structures such as the orbit, skull base and central nervous system. Approximately 75% present as locally advanced tumours of stage III and IV [2,7]. Sinonasal undifferentiated, adenoid cystic and squamous cell carcinoma have a higher tendency of neural invasion [9].

This case highlights the variation of its presentation and a positive visual outcome following treatment with corticosteroid. The patient presented with sudden visual loss upon waking up, a rare presentation for sinonasal carcinoma as usually they present with a more gradual onset. Her other ocular complaint was the deficiency of emotional tears, suggesting more extensive intracranial disease involvement, in particular the parasympathetic supply to the lacrimal gland. In our case, infiltration may have occurred at the postganglionic parasympathetic secretomotor fibres as they join the maxillary nerve at the foramen rotundum, which correlated radiologically with perineural spread involving the inferior orbital fissure and foramen rotundum of the right orbit.

Early compressive optic neuropathy may be reversible by corticosteroid treatment when oedema predominantly affects the vision [6]. Further compression leads to ischaemia, disruption of axoplasmic flow, demyelination and eventually causing optic atrophy and irreversible visual loss even with corticosteroid treatment [10]. This patient presented with acute severe visual loss. Thus, the vision improvement following corticosteroid therapy suggests that the neurological insult on the optic nerve is still reversible as the oedema settles.

There is no standard clinical guideline or prospective randomized trial on the usage of corticosteroid for compressive optic neuropathy. Decompression can be done either medically using high dose intravenous corticosteroid, or surgically by tumour debulking. Pulse-steroid therapy has been known to relieve optic neuropathy of inflammatory and compressive type [11,12]. By reducing tissue oedema, immediate visual improvement can be achieved, as seen in our patient’s vision improvement after corticosteroids. Slow steroid tapering is essential as it acts as an adjunct to chemotherapy and radiotherapy [6]. In a case series on compressive optic neuropathy secondary to metastatic prostate cancer, treatment with high dose methylprednisolone followed by radiotherapy was given with complete recovery of vision in one patient and satisfactory outcome in another two patients [10]. It is essential that the diagnosis is confirmed before steroids commencement, especially to rule out fungal infection which may worsen the patient’s condition. Based on case reports of patients who abruptly stopped their steroid, the vision usually deteriorates and although retreatment with pulse IV methylprednisolone was given, no further improvement in vision was obtained [6].

The approach for sinonasal carcinoma with orbital involvement is multimodal therapy, including surgery, radiotherapy and chemotherapy. The standard of care is complete surgical resection with postoperative radiotherapy [8]. Preoperative radiotherapy may be used to shrink the tumour, reducing damage to vital structures such as the eye. The aim is to complete tumour resection whilst preserving the eye function [1]. Corticosteroid treatment may be used to temporarily relieve the optic nerve compression whilst awaiting commencement of definitive therapy.

## Conclusions

Acute visual loss is an uncommon presentation of sinonasal carcinoma causing compressive optic neuropathy. Early treatment with corticosteroid may be beneficial in relieving the compression and improve vision in the initial phase. As an adjunct therapy, treatment with corticosteroid should be tapered slowly upon commencement of definitive therapy including chemotherapy, radiotherapy and surgery.
